# Impact in Vocal Quality in Partial Myectomy and Neurectomy Endoscopic of Thyroarytenoid Muscle in Patients with Adductor Spasmodic Dysphonia

**DOI:** 10.1016/S1808-8694(15)30066-5

**Published:** 2015-10-19

**Authors:** Domingos Hiroshi Tsuji, Fernanda Silveira Chrispim, Rui Imamura, Luiz Ubirajara Sennes, Adriana Hachiya

**Affiliations:** a Associate Professor of Otolaryngology - FMUSP, Atending physician at the Department of Otolaryngology - University Hospital - Medical School of the University of São Paulo (FMUSP).; b Otolaryngologist.; c Colaborator Professor of Otolaryngology FMUSP, Atending physician at the Department of Otolaryngology - University Hospital - Medical School of the University of São Paulo (FMUSP).; d Associate Professor of Otolaryngology - FMUSP.; e Otolaryngologist. Department of Otolaryngology - University Hospital - Medical School of the University of São Paulo.

**Keywords:** Spasmodic dysphonia, Botulinum toxin, Dystonia VHI

## Abstract

Impact in vocal quality in partial myectomy and neurectomy endoscopic of thyroarytenoid muscle in patients with adductor spasmodic dysphonia the adductor spasmodic dysphonia is a severe vocal disorder characterized by muscle laryngeal spasms during speech, producing phonatory breaks, forced, strained and strangled voice. Its symptoms come from involuntary and intermittent contractions of thyroarytenoid muscle during speech, which causes vocal fold strain, pressed one against another and increased glottic resistance. **Aim:** report the results in the impact in vocal quality in neurectomy of the thyroarytenoid branch of the inferior laryngeal nerve by endoscopic route associated with partial myectomy of the thyroarytenoid muscle with co2 laser. **Material and method:** the surgery was done in 07 patients (06 females and 01 male), aged 22 to 75, with adductor spasmodic dysphonia. They were submitted to vhi (voice handicap index) before and after surgery. **Results and conclusions:** the vocal improvement was obtained in all studied patients, deterioration in vocal quality after surgery was not noticed. There was evident difference in the vhi before and after surgery. This surgical thecnique proved to be efficient and innovative in the treatment of adductor spasmodic dysphonia.

## INTRODUCTION

Adduction spasmodic dysphonia is a vocal disorder characterized by laryngeal muscle spasms during speech, causing phonatory breaks, forced, strained and strangled voice^1^, ^2^. This disorder, classified as central origin focal dystonia, of unknown etiology, remains one of the most difficult to treat dysphonias^3^. Its symptoms come for intermittent and involuntary contractions of thyroarytenoid muscles during speech^1^, ^4^, resulting in tense vocal folds, pressed one against the other and increase in glottic resistance^1^, ^4^.

It usually starts in the third decade of life, and it happens more frequently to women. Vocal quality worsens, usually in response to stress, and there may be some improvement with the use of sedatives such as alcohol and benzodiazepins^5^. It may have a negative impact on the patient’s life quality and cause social isolation^6^.

Along the history of laryngology, different types of treatment have been proposed for this disease, such as surgeries on the larynx innervation^7^, ^8^, ^9^, ^10^, a technique of chemical denervation of the thyroarytenoid muscle by the application of botulin toxin^11^, surgery on the larynx framework, such as types II^3^, ^12^ and III^13^, ^14^ thyroplasties, partial myectomy of the thyroaritenoid muscle with cold forceps, as proposed by Fukuda (1996)^15^ and CO_2_ laser surgery, proposed by Garcia Tapia (2000)^16^. Vocal therapy is not efficient n the treatment of spasmodic dysphonias, specially in moderate and severe cases^17^.

Currently, the injection of butulin toxin is considered a first choice method by most laryngologists^18^, ^19^, ^20^. This is maily due to the excellent vocal results attained and the ease of application. Among the disadvantages of such treatment we list: the need for reaplication every three to four months, vocal instability observed in the beginning and end of its action, lack of uniformity in the results, possibility of antibody production that may neutralize its effectiveness^21^, ^22^, high cost of medication and the need for proper equipment used in its application^20^.

In face of short lasting results from partial thyroarytenoid myectomy seen by the author and published by Tsuji et al (2002)^23^, a new technique has been developed, in which we perform an endoscopic neurectomy of the thyroaritenoid branch of the inferior laryngeal nerve, together with partial myectomy of the thyroaritenoid muscle with a CO_2_^25^ laser. The goal of the present paper is to present preliminary results on vocal quality impact caused by this new techninque for the treatment of adduction spasmodic dysphonia treatment.

## MATERIALS AND METHODS

From October 2001 to May 2005, the same surgeon (DHT), performed 7 endoscopic neurectomies of the thyroaritenoid branch of the inferior laryngeal nerve, together with partial myectomy of the thyroaritenoid muscle with a CO_2_ laser in patients with adduction spasmodic dysphonia.

Patient selection criteria for surgery were: 1) adduction spasmodic dysphonia diagnosis carried out by a team of otolaryngologists and speech therapists, 2) prior clinical improvement with the use of botulin toxin in the thyroaritenoid muscle, 3) patient’s choice to perform surgery as a definitive treatment, 4) patients’ informed consent to perform the surgery.

Demographic data such as patients’ age and gender were obtained, together with a history of past treatments for that ailment.

Participants answered a questionnaire applied by one of the authors in the pre and postoperative, in which the voice impact on the life of these patients was assessed. The questionnaire used was the VHI (attachment 1), adapted to Portuguese, with 30 items. Each answer is scored from 0 to 4, and the final score varies from 0 to 120, and 120 points represents the maximum value of vocal involvement perception. At the end of VHI, the patients were asked about the percentage of improvement after surgery, and the answer was graded from 0 to 100%.

## SURGICAL TECHNIQUE

Surgery is performed under general anesthesia, orotracheal intubation and microscopic visualization. We used a conventional suspension laryngoscope, used during conventional phonomicrosurgery to expose the glottic region.

### 1st Step: Thyroaritenoid muscle partial myectomy

We do a partial myectomy of the thyroaritenoid muscle, vaporizing the lateral portion of the vocal fold with a CO_2_ laser (model 20C Sharplan), set at an intensity of 3.5 watts, in continuous superpulse mode, connected to a model M 900 DF-Vasconcelos surgical microscope, with a 400mm objective lens and straight eyepiece with a 12.5x lens. The vaporization medial limit corresponds to the line that lies approximately at 1mm lateral to the transitional line between the vibratory pars of the vocal fold and the floor of the laryngeal ventricle. We vaporize as much as possible laterally with the laser beam, sometimes even reaching the internal pericondrium of the thyroid cartilage. As anterior limit we take the internal pericondrium of the thyroid cartilage located laterally to the anterior vertex of the ventricle and, as posterior limit we use the region immediately anterior to the posterior vertex of the laryngeal ventricle. The inferior limit is determined by tactile and visual estimates by the surgeon, who should try to vaporize the whole lateral thickness of the thyroaritenoid muscle, corresponding to 3mm to 5mm of depth.

Attachment 1 **VHI (VOICE HANDICAP INDEX)**

0 = NEVER 1 =ALMOST NEVER 2 =SOMETIMES 3 =ALMOST ALWAYS 4 =ALWAYS

PART I

1-Do people feel it difficult to understand your voice?

0 1 2 3 4

2-Do people feel it difficult to understand you in noise enviroments?

0 1 2 3 4

3-Do your family members have difficulty in understanding you when you call them within the house?

0 1 2 3 4

4- Do you use the telephone less often than you wish you could?

0 1 2 3 4

5- Do you avoid groups of people because of your voice?

0 1 2 3 4

6-Do you talk less to friends, neighbors and relatives because of your voice?

0 1 2 3 4

7- Do people ask you to repeat what you had just said when talking to you face to face?

0 1 2 3 4

8-Does your voice restrict your social and personnal life?

0 1 2 3 4

9-Do you feel excluded from discussions because of your voice?

0 1 2 3 4

10-Did your voice problem cause you to lose your job?

0 1 2 3 4

PART II

1-Do you feel short of breath when you talk?

0 1 2 3 4

2-Does your voice vary along the day?

0 1 2 3 4

3-Do people ask: What is wrong with your voice?

0 1 2 3 4

4-Does your voice seems dry or chiada?

0 1 2 3 4

5-Do you need to force yourself in order to produce your voice?

0 1 2 3 4

6-Is your voice clarity unpredictable?

0 1 2 3 4

7-Do you try to change your voice to sound different?

0 1 2 3 4

8-Do you strain yourself in order to speak?

0 1 2 3 4

9-Does your voice gets worse at the end of the day?

0 1 2 3 4

10- Does your voice fail in the middle of a conversation?

0 1 2 3 4

PART III

1-Does your voice quality stress you when you talk to people?

0 1 2 3 4

2-Are people irritated by your voice?

0 1 2 3 4

3-Do you think other people do not appreciate your voice problem?

0 1 2 3 4

4-Does your voice bother you?

0 1 2 3 4

5-Does your voice make you less sociable?

0 1 2 3 4

6-Do you feel impaired by your voice problem?

0 1 2 3 4

7-Do you feel annoyed when people ask you to repeat what you had just said?

0 1 2 3 4

8-Do you feel embarassed when they ask you to repeat what you had just said?

0 1 2 3 4

9-Does your voice make you feel incompetent?

0 1 2 3 4

10-Are you ashamed of your voice problem?

0 1 2 3 4

### 2nd Step: Neurectomy of the thyroaritenoid branch of the inferior laryngeal nerve

**Figure 1:**
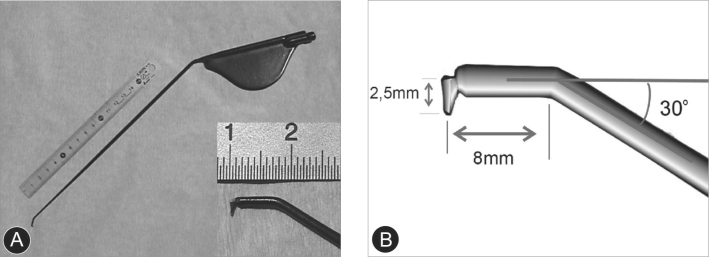
(a) Tip of the electrical scalpel; (b) Instrument angle and dimensions.

Using the tip of the electric scalpel (its shape and size are presented on Figures 1a and 1b), connected to an electrocautere (SS-601MC model of the WEM brand, set for cutting at 10.0 of intensity), we cut the thyroaritenoid branch of the inferior laryngeal nerve. The scalpel is introduced at the myectomy posterior limit, with its end initially turned posteriorly. After that we turn the scalpel in its own axis, between 45 and 90 degrees, in such a way as that the electrocautery tip is laterally positioned towards the cartilage pericondrium and allows it to reach the nerve. Following that, we perform vertical up and down movements with the electrocautere in an attempt to fish and section the nerve by electrocoagulation in order to guarantee the effectiveness in the nerve sectioning.

Surgery is performed in both vocal folds.

## RESULTS

Patients’ages varied between 22 and 75 years, with an average of 49.1 years. There were 6 females and 1 male. The average follow up time was of 23.7 months.

Except for patient^1^, all the others had received many applications of botulin toxin. They chose surgical treatment because they were not pleased with the vocal instability brought about by the botulin toxin and also for having the possibility of a definitive improvement in their clinical symptoms.

Vocal improvement was achieved in all patients, and only in one patient (patient 3) was necessary to perform a surgical correction because of vocal fold arching, in whom the right-side sterno-hyoid muscle was transposed to the paraglottic space, according to Su et al.^26^, in order to improve glottic closure. There was no vocal quality deterioration along the pos-operative period, even in the first patients who underwent the technique, and they all have close to four year follow ups.

The procedure success was seen by the degree of patient satisfaction, with improvement in vocal quality and return to social activities. Table 1 depicts the results. There was a clear difference in VHI before and after surgery, when patients reported important functional, physical and emotional voice improvement after surgery. Most patients report over 70% of subjective improvement in voice quality after surgery, and this allows them to return to their daily activities. One patient could not be found, and therefore she did not answer the VHI after surgery.

**Table 1:** VHI (Voice Handicap Index) scores per patient, carried out on August 2005.

Patient	Age (years)	Gender	Profession	Preoperative VHI	Postoperative VHI	Variation	Subjective voice improvement	Time after surgery (months)
1	44	F	Seamster	110	1	109	80%	46
2	30	F	Cachier	107	16	91	80%	46
3	54	F	Secretary	101	66	35	70%	20
4	22	F	Secretary	95	24	71	70%	41
5	67	M	Priest	97	14	83	95%	20
6	52	F	Secretary	102	20	82	80%	04

## DISCUSSION

Adduction spasmodic dysphonia is a severe disease, causing forced, strained and strangled voice, with breaks in its sonority, that may lead to an incapacity in oral communication that is difficult to treat.

In 1976, Dedo was the first to perform surgical treatment through the sectioning of the recurrent nerve, causing unilateral vocal cord paralysis. However, other studies performed^27^, ^28^, ^29^, ^30^, ^31^, ^32^ showed that some patients had recurrencies of their vocal disorder after surgery.

In our settings, the injection of botulin toxin was introduced by Tsuji^33^ and remains the first choice method to treat adduction spasmodic dysphonia^19^, ^33^. In some cases, surgery has been chosen as definitive treatment. Myectomy together with neurectomy of thyroarytenoid branch of the inferior laryngeal nerve is an alternative to the use of botulin toxin, because it is an easy to perform procedure, the endoscopic via is the access needed, it requires short surgical time, has little complication potential and offfrs the possibility of bearing definitive results.

However, the different therapeutic modes have shown that voice may be reasonably restored to a quality standard, if not totally normal, better than it was prior to surgery, since one achieves certain inactivity of the thyroaritenoid muscle, or a certain reduction on the degree of glottic closure, and this happens through surgery of the larynx framework. Both the recurrent nerve sectioning technique and the isolate thyroaritenoid myectomy require cold forceps (Fukuda^15^). CO_2_ laser (described by Garcia Tapia^16^), and botulin toxin injection fail when the thyroarytenoid muscle resumes its total contractility (as we have in the case of toxin injection) or partial (in the case of the DEDO procedure, and, very likely, in the myectomy). The mechanisms responsible for the thyroarytenoid muscle functional recovery, even if partial, after the aforementioned surgical approach are still not clear, but they are probably related to the reinervation of denerved muscle fibers.

When we associate the neurectomy of the thyroarytenoid branch of the inferior laryngeal nerve to thyroarytenoid muscle myectomy, we aim at preventing, or at least making it difficult, the reinervation of the remaining muscle from myectomy.

VHI was proposed by Jacobson et al.^34^ and describes the physical, emotional and functional impact voice has on the life of these people. In the present study, we used the questionnaire in patients with adduction spasmodic dysphonia who underwent thyroarytenoid muscle myectomy together with the neurectomy of the nerve of the same name shows clear and long lasting improvement in voice when we considere the three aspects surveyed, with patient social and economical reintegration.

## CONCLUSION

The neurectomy of the thyroarytenoid branch of the inferior laryngeal nerve together with partial myectomy of the thyroarytenoid muscle represents an efficient and innovative surgical technique for the treatment of adduction spasmodic dysfunction, and it may be a therapeutic option for those patients who seek a definitive improvement of their problem.
